# Effect of 12-week intermittent calorie restriction compared to standard of care in patients with nonalcoholic fatty liver disease: a randomized controlled trial

**DOI:** 10.1186/s13063-023-07444-4

**Published:** 2023-08-02

**Authors:** Han Ah Lee, Hyeyoung Moon, Yuri Kim, Hye Ah Lee, Hwi Young Kim

**Affiliations:** 1 Department of Internal Medicine, College of Medicine, Ewha Womans University, 1071 Anyangcheon-Ro, Yangcheon-Gu, Seoul, 07985 Republic of Korea; 2 Nutritional Science and Food Management, Ewha Womans University, Seoul, Republic of Korea; 3 Clinical Trial Center, Ewha Womans University Seoul Hospital, Seoul, Republic of Korea

**Keywords:** Nonalcoholic fatty liver disease, Nonalcoholic steatohepatitis, Intermittent calorie restriction, Steatosis, Fibrosis, MRI-proton density fat fraction, Elastography, Weight reduction, Dietary therapy

## Abstract

**Background:**

Nonalcoholic fatty liver disease (NAFLD) is the most common cause of chronic liver disease. NAFLD can result in various complications. Owing to the lack of effective pharmacological therapies, lifestyle modifications are the cornerstone treatment for NAFLD. However, there has been no recommendation for a specific dietary therapy. Because no significant effects have been observed in previous studies. Intermittent calorie restriction (ICR) consists of alternating phases of extreme energy restriction and regular energy intake. Recent studies have demonstrated a significantly higher reduction in liver fat content in the ICR group than in the standard of care (SOC) or continuous calorie restriction groups in patients with NAFLD. However, critical weaknesses limit the broader application of ICR in clinical practice; those are a lack of appropriate assessment tools, different cutoffs of body mass index (BMI) used to define obesity, and different food portions. Thus, we report a protocol for a prospective, randomized controlled trial. The trial will evaluate the effect of 12-week ICR on improving liver fat content in NAFLD patients (Nonalcoholic Fatty Liver Disease-Intermittent Calorie Restriction [FLICR]).

**Methods:**

We will include adult (19–75 years) NAFLD patients. NAFLD will be diagnosed by histologic assessment or magnetic resonance imaging-proton density fat fraction (MRI-PDFF) ≥ 8%. A total of 72 patients will be classified according to BMI (obese group: BMI ≥ 25 kg/m^2^ [*n* = 36] and non-obese group: BMI < 25 kg/m^2^ [*n* = 36]). Participants will be followed up for 24 weeks. Participants will be randomly assigned to one of the two groups: the SOC or ICR group. The primary objective will be the change in liver fat content measured using MRI-PDFF from baseline to 12 weeks.

**Discussion:**

This FLICR study may provide clinical evidence on ICR in the treatment of NAFLD in both obese and non-obese patients. The use of ICR in patients with NAFLD will improve the clinical outcomes of patients facing a shortage of effective medical therapy.

**Trial registration:**

This trial was registered at the United States National Library of Medicine (NLM) at the National Institutes of Health. ClinicalTrials.gov NCT05309642. Registered on April 4, 2022.

**Supplementary Information:**

The online version contains supplementary material available at 10.1186/s13063-023-07444-4.

## Introduction

### Background and rationale

Nonalcoholic fatty liver disease (NAFLD) is the most common cause of chronic liver disease worldwide, affecting approximately 25% of the world’s population [1]. In Korea, the prevalence of NAFLD has been reported from 20 to 30% [2]. The broad spectrum of NAFLD encompasses simple hepatic steatosis to nonalcoholic steatohepatitis (NASH), which can progress to cirrhosis in 15–20% of affected patients, as well as the development of hepatocellular carcinoma (HCC) and death [3, 4]. NAFLD is an independent risk factor for the development of various extrahepatic diseases, including cardiovascular disease (CVD) and malignant tumors [2].

Therefore, proper and active management is required to reduce intrahepatic inflammation and fibrosis and to treat comorbid metabolic diseases in patients with NAFLD. However, because effective pharmacological therapies for NAFLD have not yet been approved, non-pharmacologic therapies including weight reduction through dietary control and exercise are the cornerstone treatment for both obese and non-obese NAFLD patients, based on the results of previous studies [5–7].

Although recent studies have evaluated the association between specific nutrients or dietary habits including low carbohydrate/high-fat diet or intermittent fasting and the development or progression of NAFLD, these studies have been conducted on only a small number of patients, and few have shown significant improvement in hepatic inflammation or fibrosis [8–12]. Intermittent calorie restriction (ICR), comprising phases of extreme energy restriction and regular energy intake, is a novel dietary approach for NAFLD treatment [13]. Significantly higher reductions in liver steatosis with ICR than with the standard of care (SOC) or continuous calorie restriction (CCR) have been reported in NAFLD patients [14–17]. Although promising findings have been reported in previous studies of 6–12 weeks of dietary intervention, the lack of appropriate assessment tools, different cutoffs of body mass index (BMI) used to define obesity, and different food portions between studies are the major limitations [14–17].

### Objectives

Therefore, we hypothesized that ICR would be effective in reducing liver fat content measured using magnetic resonance imaging-proton density fat fraction (MRI-PDFF) compared to SOC in patients with NAFLD. Finally, we developed a prospective, randomized controlled trial (RCT) to evaluate the effect of 12-week ICR on improving liver fat content in NAFLD, stratifying patients into obese and non-obese groups.

### Trial design

The Nonalcoholic Fatty Liver Disease-Intermittent Calorie Restriction trial was designed as a prospective, two-arm, open-label RCT to investigate the efficacy of 12-week ICR in reducing liver fat content in NAFLD patients.

## Methods: participants, interventions, and outcomes

### Study setting

The study will recruit 72 patients who will be followed up actively according to the protocol for 24 weeks. Patients will be classified according to their BMI (obese group: BMI ≥ 25 kg/m^2^ [36 patients] and non-obese group: BMI < 25 kg/m^2^ [36 patients]). An enrollment period of 12 months is expected.

### Eligibility criteria

The following are the inclusion criteria:

1. NAFLD diagnosed by (1) histologic assessment with a fat accumulation of > 5% of the liver’s weight on biopsy or (2) radiologic assessment with MRI-PDFF ≥ 8%
2. Age between 19 and 75 years
3. Capability to understand the study and the individual consequences of participation
4. Signed and dated declaration of agreement in the forefront of the study

The following are the exclusion criteria:

1. Daily alcohol consumption > 30 g in men and > 20 g in women
2. Other causes of chronic liver disease (hepatitis B, C, D, and E virus infection; human immunodeficiency virus; autoimmune diseases; chronic cholestatic liver disease; drug-induced liver injury; hereditary hemochromatosis; Wilson disease; and α-1-antitrypsin deficiency)
3. Liver cirrhosis
4. Hepatocellular carcinoma
5. Medications that cause liver disease or secondary NAFLD (e.g., tamoxifen, systemic corticosteroids, methotrexate, tetracycline, estrogens, and valproic acid)
6. Changes in body weight > 5% in the last 3 months
7. Intake of medical treatment for NAFLD/NASH in the last 6 months (except vitamin E)
8. Diabetes
9. Pregnancy
10. Patients after organ transplantation
11. Missing or lacking consent capability

### Who will take informed consent?

The investigators will be obtaining the informed consent from each study participant. Each participant must have voluntarily given informed consent and signed by the patient and an investigator before any study-specific procedures are initiated.

### Additional consent provisions for the collection and use of participant data and biological specimens

On the consent form, participants will be asked if they agree to the use of their data should they choose to withdraw from the trial. Participants will also be asked for permission for the research team to share relevant data with people from the universities taking part in the research or from regulatory authorities, where relevant. Therefore, all those additional tests will be performed only after obtaining informed consent, based on each participant’s voluntary participation and agreement to those procedures.

### Interventions

#### Intervention description

##### Active comparator (ICR arm)

On two non-consecutive days per week, participants in the ICR (5:2 diet) group will be instructed to consume 500 kcal/day for women and 600 kcal/day for men. Recipes will be provided with suggestions for meals that do not exceed calorie restrictions. For the remaining 5 days of the week, they will receive instructions and recipes that follow the Korean Dietary Reference Intakes, with an intake limit of 2000 kcal/day for women and 2500 kcal/day for men. The percentage of energy (E%) from the different macronutrients in the recipes will be 45–60 E% carbohydrates, 25 E% fat, and 10–20 E% protein.

##### Placebo comparator (SOC arm)

The SOC group will receive 80% of the standard calories (1200–1500 kcal/day or reducing 500–1000 kcal/day from standard calories). They will receive individualized guidance from a hepatologist on how to choose a healthy diet, reduce the intake of sweets and saturated fatty acids, increase sources of unsaturated fat, avoid large portions, and regularly eat three meals per day. Sample meal plans by calorie intake are presented in Supplementary materials.

#### Criteria for discontinuing or modifying allocated interventions

Patients may be discontinued from the study at any time if, in the opinion of the investigator, it is medically necessary or if it is an expressed wish of the patient. The patients are free to discontinue their participation in the trial at any time.

#### Strategies to improve adherence to interventions

Study visits will be performed at baseline and every week during the intervention period (telephone consultation with 24-h recall or visit to the clinic with a 3-day diet diary).

#### Relevant concomitant care permitted or prohibited during the trial

At baseline visit (0 weeks), the exercise practitioner will conduct exercise consultation and educate enrolled participants to do at least moderate-intensity exercise for more than 30 min more than 3 times per week throughout the trial period.

#### Provisions for post-trial care

N/A

### Outcomes

The primary objective is to evaluate the impact of ICR on liver steatosis measured using MRI-PDFF (time frame: baseline and 12 weeks), and the outcome of interest is a change in the MRI-PDFF value of at least 30% with ICR. Secondary objectives include changes in liver fibrosis by MRE (time frame: baseline and 12 weeks); anthropometrics including BMI (kg/m^2^) (time frame: baseline, 12 and 24 weeks); BCA measured using InBody (time frame: baseline, 12 and 24 weeks); VAT measured using fat computed tomography (CT) (time frame: baseline, 12 and 24 weeks); QOL score measured using Chronic Liver Disease-NAFLD Questionnaire (CLDQ) (time frame: baseline, 12 and 24 weeks); hormones including leptin, adiponectin, ghrelin, GLP-1, apelin, chimerin, and osteopontin (time frame: baseline and 12 weeks); liver metabolites including diglycerides, fatty acid, phosphatidylethanolamines, phosphatidylcholines, methionine, S-adenosyl-l-methionine, and methylthioadenosine (time frame: baseline and 12 weeks); and gut microbiota analyzed with 16S rRNA gene sequencing (time frame: baseline and 12 weeks). Polymorphisms of PNPLA3, TM6SF2, TM4SF5, SREBF2, MBOAT7-TMC4, HSD17B13, and adenine insertion (A-INS) will also be analyzed using next-generation sequencing (NGS). The objectives of this study are summarized in Table 1.

**Table 1:** Objectives of the Nonalcoholic Fatty Liver Disease-Intermittent Calorie Restriction (FLICR) study

	Study time points (weeks)			
	Intervention period			Maintenance period
	0	6	12	24
**Primary objective**				
Change in liver steatosis by MRI-PDFF	■		■	
**Secondary objectives**				
Change in liver fibrosis by MRE	■		■	
Change in anthropometrics	■		■	■
Change in BCA by InBody	■		■	■
Change in VAT by fat CT	■		■	■
Change in quality of life score by CLDQ	■		■	■
Change in hormone	■		■	
Change in liver metabolites	■		■	
Change in gut microbiota by 16S rRNA gene sequencing	■		■	
Polymorphism of SNPs using NGS	■			

*MRI* Magnetic resonance imaging, *PDFF* Proton density fat fraction, *MRE* Magnetic resonance elastography, *BCA* Body composition analysis, *VAT* Visceral adipose tissue, *CT* computed tomography, *CLDQ* Chronic Liver Disease-NAFLD Questionnaire, *SNP* Single nucleotide polymorphism, *NGS* Next-generation sequencing

### Participant timeline

**Fig. 1:**
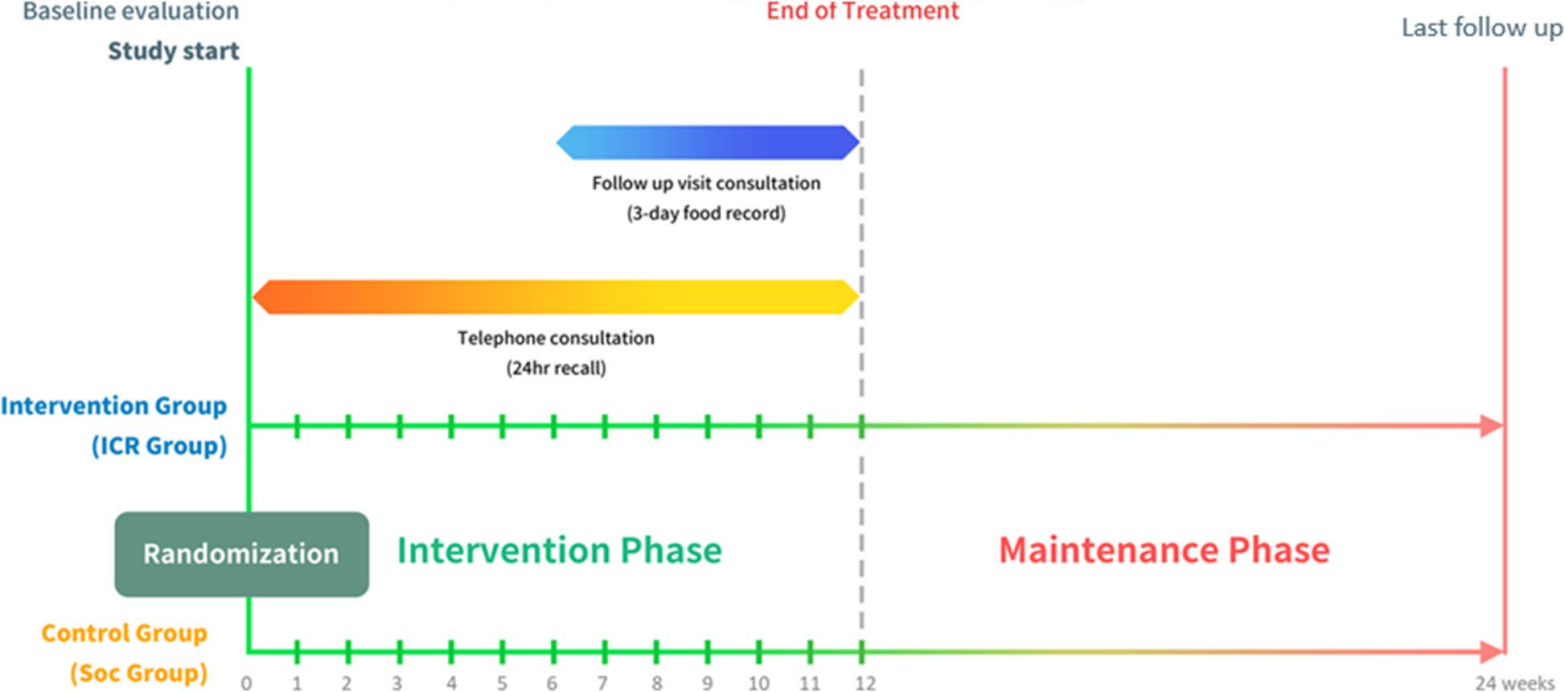
Study timeline. ICR, intermittent calorie restriction; SOC, standard of care

The patients will be followed up for 24 weeks (Fig. 1). Detailed dietary consultation will be conducted by a nutritionist at the baseline visit, and each participant will be provided with a written summary of the dietary advice. The dietary intervention will be performed during the first 12 weeks, and 12 weeks hereafter is the maintenance period, and patients will be followed up at the end of the study period. The schedule of the assessments of the study procedures is summarized in Table 2.

**Table 2:** The schedule of enrollment, interventions, and assessments

Time point	Study period					
	**Enrollment**	**Allocation**	**Post-allocation**			**Close-out**
	*** − t***_***1***_	***0***	***0 weeks***	***6 weeks***	***12 weeks***	***24 weeks***
**Enrollment**						
**Eligibility screen**	X					
**Informed consent**	X					
**Screening**	X					
**Allocation**		X				
**Interventions**						
*** [ICR]***			X	X	X	
*** [SOC]***			X	X	X	
**Examinations**						
***MRI-PDFF, MRE***			X		X	
***Anthropometrics***			X	X	X	X
***Blood sample***			X		X	X
***Stool, urine sample***			X		X	X

Follow-up at 12 weeks (end of treatment) will include the following mandatory tests: assessment of liver fat by MRI-PDFF, liver fibrosis by magnetic resonance elastography (MRE), anthropometrics, body composition analysis (BCA), visceral adipose tissue (VAT), food intake, quality of life (QOL), liver metabolites, microbiome, and hormones (leptin, adiponectin, ghrelin, GLP-1, apelin, and osteopontin). Follow-up will be terminated at the end of the maintenance period (24 weeks), and anthropometrics, BCA, VAT, food intake, and QOL will be assessed. All study participants, except those who withdraw consent for the study, will be followed up for the study period.

### Sample size

In a previous study, the percentage of participants who reached the endpoint of a relative reduction in liver fat content > 30% measured using MRI-PDFF after 12 weeks was 80% in the ICR group and 29% in the control group [17]. Based on this assumption, 72 participants (36 in the obese group and 36 in the non-obese group) are planned to be randomized into the two treatment groups in a 1:1 ratio (ICR:SOC) to achieve 80% power for superiority comparison (Fisher’s exact test) of the primary objective between the two treatment groups, with a two-sided type I error of 5% and allowing for a 10% dropout rate. G*Power 3.1.9.4 was used to calculate the sample size.

### Recruitment

The FLICR trial will recruit patients from Ewha Womans University Mokdong Hospital. This study will only recruit Korean patients. The Department of Nutritional Science and Food Management, Ewha Womans University, will collaborate with this study. The randomized allocation will be conducted at Ewha Womans University Mokdong Hospital. Eligible patients will be screened by the principal and sub-investigator (hepatologists) and will undergo screening to investigate whether they fulfill the eligibility criteria and none of the exclusion criteria for the study.

### Assignment of interventions: allocation

For eligible patients, the principal or sub-investigator will complete the patient enrollment form and enroll the patients. Patients will be randomly assigned to receive ICR or SOC by the enrolling investigator via a computer-generated randomization sequence using block randomization (block size = 6) with a 1:1 ratio and stratification by BMI (≥ 25 and < 25 kg/m^2^). After the participants have been assigned to the ICR or SOC group through the randomization procedure, they will be informed by the enrolling investigator.

### Assignment of interventions: blinding

This study is an open-label trial, where both the researchers and participants are aware of the treatment or medication they are receiving. The radiologists who assess the MRI results are blinded to the patient assignment group and clinical information.

### Data collection and management

#### Data management

Investigators will enter the data required by the protocol into the case report forms (CRF). The principal investigator is responsible for assuring that data entered into the CRF is complete and accurate, and that entry is performed in a timely manner. The signature of the investigator will attest to the accuracy of the data on each CRF. All data management activities will be completed prior to the final closure of the database.

#### Confidentiality

All patient data collected and processed for the purposes of this study will be managed by the investigators with adequate precautions to ensure the confidentiality of the data and in accordance with applicable national laws and regulations on personal data protection. No patient-identifiable data will be obtained. In any presentation of the results of this study, at meetings, or in publications, the patients’ identities will remain confidential. In all activities, the General Data Protection Regulation will be followed to ensure the protection of sensitive personal information, and the study will be performed in accordance with the World Health Organization guidelines for good clinical practice.

#### Plans for collection, laboratory evaluation, and storage of biological specimens for genetic or molecular analysis in this trial/future use

The collected samples will be stored at Ewha Womans University Mokdong Hospital and sent to an analytical institution for analysis after the intervention and maintenance period is completed.

## Statistical methods

### Statistical methods for primary and secondary outcomes

Descriptive statistics will be computed for all variables: mean and standard deviation for continuous variables, unless stated otherwise, and frequency and percentage for categorical variables. Intention-to-treat will be performed mostly. Comparisons will be made within groups (post- vs. pre-intervention for each group) and between groups (ICR vs. SOC). Independent and paired *t*-tests will be used to compare continuous variables between and within groups, respectively. To estimate the pre/post-values and changes within and between the diets for the primary and secondary outcomes, a linear mixed model will be used. SPSS version 23 (SPSS Inc., Chicago, USA) will be used for statistical analysis. All two-sided *P* value < 0.05 will be considered as statistically significant.

### Interim analyses

We will not perform interim analysis to preserve statistical power and data integrity and to minimize bias and data-dependent decisions. If significant harm or safety issues arise during the trial, the study may be halted to protect the participants. The study will be also stopped if ethical concerns, such as the emergence of a new standard of care, develop.

### Methods for additional analyses (e.g., subgroup analyses)

Subgroup analyses will be performed according to the obese and non-obese groups.

### Methods in analysis to handle protocol non-adherence and any statistical methods to handle missing data

The main analyses will be performed based on the intention-to-treat population, and missing data will be assumed to be missing at random.

### Plans to give access to the full protocol, participant-level data, and statistical code

The datasets analyzed during the current study and statistical code are available from the corresponding author upon reasonable request, as is the full protocol.

### Oversight and monitoring

#### Composition of the coordinating center and trial steering committee

There will always be a group running the trial day-to-day and providing organizational support and knowing how often they will meet, plus information on other committees providing oversight such as a Trial Steering Committee.

#### Composition of the data monitoring committee, its role, and reporting structure

An independent data monitoring committee will be established with two individuals from the Department of Biostatistics, Ewha Womans University Mokdong Hospital. The management team will observe the progress and data monthly by mail and web conferencing. If the monitoring committee decides that on-site monitoring is necessary, members will visit the site for face-to-face monitoring.

#### Adverse event reporting and harms

All AEs during the study will be recorded with the following data: date of onset and date of completion (if applicable), severity of AEs, investigator’s view on the relationship to dietary intervention, taken actions on AEs, treatment of the AEs, cause of the event (if known), and outcome. The AE intensities will be graded according to the National Cancer Institute’s Adverse Event Common Terminology version 4.03.

#### Frequency and plans for auditing trial conduct

The project management group will meet to review trial conduct every month, and the trial steering group and the independent data monitoring and ethics committee will meet to review the trial conduct every 3 months throughout the trial period.

## Discussion

NASH can progress to cirrhosis, HCC, and death and is an increasing indication for liver transplantation [4]. Because of the lack of effective pharmacological therapies, lifestyle modifications are the mainstay of treatment for NAFLD [5]. Although no obvious effect of specific dietary therapies has been observed in previous studies, reduced energy intake resulted in weight loss, decreased intrahepatic fat content, decreased liver enzyme levels, and decreased insulin resistance in several RCTs [8, 9, 18]. Therefore, the current recommendations are limited to the restriction of total daily calorie intake within 1500–1800 kcal in men and 1200–1500 kcal in women or a reduction in total energy intake of > 500 kcal/day [19].

ICR, as a novel dietary approach in the treatment of NAFLD, has the two most common strategies of strict alternate-day fasting and a 5:2 diet with two self-selected days with extreme energy restriction and 5 days without calorie restriction [13, 20]. Recent studies have demonstrated a greater reduction in liver steatosis with ICR than that with SOC in NAFLD patients [13, 15, 16]. Other studies suggested that insulin concentrations and fat mass were significantly reduced with ICR compared to that with CCR, with similar amounts of net calorie intake and weight loss [14, 15]. ICR can also improve dietary compliance and reduce cardiometabolic risks [17, 20]. However, only few studies have assessed liver fat content with MRI to date [17]. In addition, active calorie restriction according to the guidelines was not performed in the control group [17].

Despite these promising findings, further studies are required to address the multiple areas of uncertainty. It remains to be elucidated whether ICR is superior to CCR at similar amounts of net energy intake with respect to a broader set of metabolic health indicators [7]. In addition, analyses of long-term maintenance of initial weight loss and metabolic outcomes with ICR are required to investigate its sustainability and practicability [8, 9] because the first systematic review of the few ICR studies that had longer than 6 months of follow-up did not show greater benefits of ICR compared with those of CCR [18].

On considering the disease burden, including hepatic and extrahepatic complications, preventive therapies to inhibit the progression of fatty liver disease to NASH are required [21]. In this prospective RCT, we will compare the effect and safety of 12-week ICR vs. SOC (counseling and inducing active calorie restriction according to the guidelines) in NAFLD treatment regarding the reduction in liver fat content, fibrosis, BMI, and body fat content. Specifically, we will assess the change in liver fat content with MRI, the most accurate modality in the quantification of liver steatosis that currently exists. In addition, further evaluation including changes in QOL, hormones, liver metabolites, gut microbiota, and genetic alterations in NAFLD patients will be performed.

Previous studies have shown decreased intrahepatic fat content with weight loss of > 5–7% and improvement of hepatic inflammation and fibrosis with weight loss of > 7–10% in obese NAFLD patients [6]. In non-obese NAFLD patients, a recent study reported that a weight loss of > 3–5% was effective in the remission of NAFLD; however, further studies are needed for validation [7]. Therefore, to evaluate the effect of ICR and the resulting weight loss in the treatment of NAFLD in non-obese patients, we will stratify the patients into two groups of obese and non-obese groups. This study will only enroll Korean patients; therefore, a BMI cutoff of 25 kg/m^2^, the standard in the Eastern Mediterranean population, was selected to define obesity.

Finally, evaluating the regression or progression of NAFLD with histology would be ideal, especially in 22 terms of diagnosis of NASH and assessment of progression/regression of NASH and/or fibrosis. Because this study is not a novel drug trial, performing a liver biopsy in the entire study participants might not be justified given that the treatments in the study are two forms of dietary intervention. In addition, the downsides of liver biopsy, such as invasiveness, costs, sampling error, and interobserver variability, were considered [22]. Nonetheless, the histological assessment will be at least performed at baseline if a participant is suspected to have NASH (particularly fibrotic NASH) [23].

In conclusion, this FLICR study may provide clinical evidence for ICR in the treatment of NAFLD in both obese and non-obese patients. The use of ICR in patients with NAFLD will improve the clinical outcomes of patients facing a shortage of effective medical therapy.

## Trial status

Recruitment of participants has begun in July 2022 and closed in January 2023.

## Supplementary Information

**Additional file 1.** Sample meal plan by calorieintake.

## Data Availability

The datasets analyzed during the current study are available from the corresponding author on reasonable request, as is the full protocol.

## Abbreviations

NAFLD: Nonalcoholic fatty liver disease
NASH: Nonalcoholic steatohepatitis
HCC: Hepatocellular carcinoma
CVD: Cardiovascular disease
ICR: Intermittent calorie restriction
SOC: Standard of care
CCR: Continuous calorie restriction
BMI: Body mass index
MRI: Magnetic resonance imaging
PDFF: Proton density fat fraction
RCT: Randomized controlled trial
MRE: Magnetic resonance elastography
BCA: Body composition analysis
VAT: Visceral adipose tissue
QOL: Quality of life
FLICR: Fatty liver and intermittent calorie restriction
CT: Computed tomography
CLDQ: Chronic Liver Disease-NAFLD Questionnaire
NGS: Next-generation sequencing
AE: Adverse event
