# Supplementary material for: Effect of 12-week intermittent calorie restriction compared to standard of care in patients with nonalcoholic fatty liver disease: a randomized controlled trial
Source: Trials. 2023 Aug 2;24:490. doi: 10.1186/s13063-023-07444-4 (PMC10394920; doi:10.1186/s13063-023-07444-4)
Supplement: Supplementary file 1 — Additional file 1. Sample meal plan by calorieintake. [file 13063_2023_7444_MOESM1_ESM.docx]

**Supplementary materials**

**Sample Meal Plan by Calorie Intake**

**1. 1200 kcal**

• Breakfast

2/3 serving of white rice

Potato soup (1/2 potato)

Steamed egg custard (55g)

Stir-fried anchovies (15g)

Kimchi

• Snack

1/3 apple

• Lunch

Cold buckwheat noodles (180g)

Grilled pork bulgogi (40g)

Cucumber salad

Young radish kimchi

• Snack

200ml low-fat milk

• Dinner

1 serving of multigrain rice

Tofu soybean paste soup (40g tofu)

Grilled saury fish (1 piece)

Lettuce salad

Radish kimchi

**2. 1,500 kcal**

• Breakfast

1 serving of white rice

Potato soup (1/2 potato)

Steamed egg custard (55g)

Stir-fried anchovies (15g)

Kimchi

• Snack

1/3 apple

• Lunch

Cold buckwheat noodles (180g)

Grilled pork bulgogi (40g)

Cucumber salad

Young radish kimchi

• Snack

200ml milk

• Dinner

1 serving of multigrain rice

Tofu soybean paste soup (40g tofu)

Grilled saury fish (1 piece)

Lettuce salad

Radish kimchi

**3. 2,000 kcal**

• Breakfast

1 serving of white rice

Potato soup (1/2 potato)

Rolled omelet (55g)

Stir-fried fish cakes (50g)

Kimchi

• Snack

1/3 apple

• Lunch

Cold buckwheat noodles (270g)

Grilled pork bulgogi (80g)

Cucumber salad

Young radish kimchi

• Snack

200ml milk

• Dinner

1 serving of multigrain rice

Tofu soybean paste soup (40g tofu)

Grilled saury fish (2 piece)

Lettuce salad

Radish kimchi

• Snack

1/2 corn cob

**4. 500 kcal**

• Type 1

Mixed grain rice 2/3 serving

Beef seaweed soup

Grilled mackerel fish (1 piece)

Stir-fried spinach

• Type 2

Chicken breast (80g)

Tomato salad

Balsamic oil (5 teaspoons)

Whole wheat bread (1 piece)

Café latte (200ml)

• Type 3

Soybean noodle soup

Boiled noodles (90g) + kelp noodles

Soybean broth (500ml)

Boiled egg (1 piece)

Cucumber, tomato

• Type 4

Greek yogurt (100g)

Granola/oatmeal (30g)

Fruits (blueberries, etc.)

Nuts (8g)

Egg (1 piece)
